# Exploring direct detection suppressed regions in a simple 2-scalar mediator model of scalar dark matter

**DOI:** 10.1140/epjc/s10052-021-09170-0

**Published:** 2021-05-10

**Authors:** Jérôme Claude, Stephen Godfrey

**Affiliations:** Department of Physics, Ottawa-Carleton Institute for Physics, Carleton University, Ottawa, K1S 5B6 Canada

## Abstract

We explore regions of parameter space that give rise to suppressed direct detection cross sections in a simple model of scalar dark matter with a scalar portal that mixes with the standard model Higgs. We found that even this simple model allows considerable room in the parameter space that has not been excluded by direct detection limits. A number of effects leading to this result have been previously noted. Our main new result explores interference effects between different contributions to DM annihilation when the DM mass is larger than the scalar portal mass. New annihilation channels open up and the parameters of the model need to compensate to give the correct DM relic abundance, resulting in smaller direct detection cross sections. We find that even in a very simple model of DM there are still sizeable regions of parameter space that are not ruled out by experiment.

## Introduction

There is considerable evidence for *Dark Matter* (DM), a type of matter in the universe which has so far only revealed itself through gravitational interactions with normal matter [1–3]. DM at most interacts very weakly with normal matter. Various means of DM interacting with normal matter have been explored; Higgs portals, e.g. [2, 4–26], vector portals, e.g. [15, 18, 27–47], and neutrino portals, e.g. [15, 18, 48–51]. Higgs portal models have been tightly constrained by experiment, leaving only small regions in the parameter space viable [40]. In particular, direct detection experiments have tightly constrained the parameter space. However, there still exist allowed regions, including regions referred to as *blind spots* which are due to cancellations in the direct detection cross section amplitudes. This has been explored in a number of papers, for example [10, 20, 21, 23, 52–57]. In addition to the blind spots mechanism, other mechanisms exist that suppress direct detection cross sections which we discuss below.

Many Higgs portal models have a second scalar that mixes with the Standard Model (SM) Higgs boson [7, 9, 10, 12, 14, 17, 20–23, 25, 26, 38, 54, 58–64]. The mechanism leading to blind spots in such models is the destructive interference between the Higgs-like scalar and the second scalar in the direct detection cross section amplitude [9, 10, 12, 17, 20, 21, 23, 26, 54, 61, 63]. Given that detecting dark matter is the focus of a broad experimental program, we felt it useful to further explore regions of the parameter space that give rise to suppressed direct detection cross sections. Our preconceived bias was that the mixing angle between the two *t*-channel exchange bosons could be tuned to create the direct detection blind spots mentioned above. However, we found that values of the mixing angle that would give rise to blind spots are for the most part ruled out by measurements of Higgs boson properties – most generally by the Higgs signal strengths, but also by the Higgs invisible width when Higgs decay to dark matter is kinematically allowed. Another mechanism that can lead to suppressed direct detection cross sections which has previously been pointed out [12, 18] is the result of a resonance effect occurring when the dark matter mass is roughly half the value of the scalar portal mass. However, there is a third mechanism that suppresses the direct detection cross section when the dark matter particle is more massive than either the Higgs boson or the portal particle.^1^ In this case, a large region of the parameter space has not been ruled out by any of the theory constraints, any experimental constraints and, more to the point of this exercise, by direct detection limits.

For the purposes of this study, we constructed a very simple toy model consisting of scalar DM and an additional scalar portal that can mix with the SM Higgs field to study direct detection suppressed regions. We use this toy model to explore effects for the simplest possible case of a scalar dark matter portal extension. There are, however, many possible variations of this simple picture that can give rise to cancellations in the direct detection cross section. An incomplete list of possibilities appearing in the literature consists of the scalar portal being replaced with a pseudoscalar portal [22, 55, 65–72], or having a complex scalar which gives rise to a second scalar portal [20, 26, 26, 57, 73, 74], a two Higgs doublet model [22, 23, 55, 63, 64, 69–71, 74–77], higher Higgs representations [78], or supersymmetric models [22, 52, 54, 79–82]. Before proceeding, we note that, given that we simply want to push the simplest of models as far as we could, we haven’t dealt with the issue of UV completeness. However, Gross et al. [20] and Huitu et al. [57] showed that they could make models very similar to ours UV complete by assuming the system is invariant under a global *U*(1) which is gauged in the UV-completeness. Other examples of similar UV complete models are [25, 26].

Our simple model has eight parameters but two are fixed to their SM values, one is fixed to give the correct DM relic abundance, and one is only weakly constrained by DM self-interactions. This leaves four independent parameters which we choose to be the scalar DM mass, the scalar portal mass, the scalar singlet vacuum expectation value, and the mixing angle between the SM Higgs scalar and the scalar portal. We scan through the parameter space and, by transforming to the Lagrangian parameters, we test that perturbative unitarity holds, that the potential is bounded from below, and that the parameters result in a consistent set of parameters for the desired properties of the model. We next fix the remaining parameter to give the correct relic abundance. With these parameter values, we test that the parameters are consistent with the Higgs boson invisible width, the Higgs signal strengths, and DM self-interaction limits. Finally, we calculate the direct and indirect detection cross sections and compare them to the experimental limits.

In Sect. 2, we give the details of our model and examine the theoretical constraints on its parameters. In Sect. 3, we describe the details of scanning the parameter space and the various experimental measurements we use to constrain parameter points, starting by fitting the DM-portal coupling to the DM relic abundance. The remaining experimental constraints are the Higgs invisible width, the Higgs signal strength, the DM self-interaction, and the DM indirect detection cross section. We then compare the points that pass all these constraints to the direct detection limits and examine the various mechanisms that lead to direct detection suppressed regions. Finally, in Sect. 4, we summarize our conclusions.

## A 2-scalar mediator model with scalar DM

We consider an extension of the Standard Model that consists of two singlet scalar fields $\varphi$ and *S*, with $\varphi$ a portal particle that mixes with the SM Higgs field and *S* the DM particle. We impose a $Z_2 \times Z_2$ symmetry on these fields so that they are odd under their respective $Z_2$’s to ensure their stability and eliminate terms in the potential odd in $\varphi$ and *S* (see for example Refs. [18, 22]). We note that the $Z_2$ imposed on $\varphi$ is spontaneously broken when $\varphi$ acquires a vev. The most general scalar potential with this symmetry is then given by

1 $$\begin{aligned} V(H, \varphi , S)= & {} -\mu _H^2 H^\dagger H + \lambda _H (H^\dagger H)^2 \nonumber \\&- {{\mu _\varphi ^2}\over 2}\varphi ^2 + {{\lambda _\varphi }\over 4}\varphi ^4 + \lambda _4 \varphi ^2 (H^\dagger H )\nonumber \\&+ {{\mu _S^2}\over 2} S^2 + {{\lambda _S}\over 4} S^4 + {{ \lambda _{\varphi \varphi SS}}\over 2} \varphi ^2 S^2 \nonumber \\&+ {{\lambda _{HH SS}}\over 2} (H^\dagger H) S^2. \end{aligned}$$

Following Refs. [18, 22], we take $\lambda _{HH SS} = 0$ so that the Standard Model complex scalar doublet *H* does not directly couple to the dark matter candidate, *S*, at tree level. This choice does not affect our conclusions, and we will discuss the consequences of not taking $\lambda _{HH SS} = 0$ in Sect. 3.7 after we present our results. This term can be generated via $\varphi$ loops and the natural size for the resulting vertex would be the product of the couplings $\lambda _{\varphi \varphi SS} \lambda _4 /(16\pi ^2)$. We assume that the vertex can be made small enough even if it requires some amount of tuning. Assuming this term is small enough, and because the DM thermally averaged annihilation cross section is typically dominated by the *s*-channel annihilation cross section and real production of $h_2$, we will find that it will not have a big effect on the relic abundance and that neglecting it will not qualitatively alter our conclusions.

We work in the unitarity gauge and shift the fields to the new minimum; $H \rightarrow (0, (v+h)/\sqrt{2})^T$ and $\varphi \rightarrow (w+\phi )$, where *v* and *w* are the vacuum expectation values (vevs) of the neutral component of *H* and $\phi$ respectively. We require that *S* does not acquire a vev so that the $Z_2$ symmetry remains unbroken and *S* is stable. With this substitution, we then minimize the resulting potential $V(h, \phi , S)$ with respect to the scalar fields and obtain $\mu _H^2=\lambda _H v^2+\lambda _4w^2$ and $\mu _\varphi ^2=\lambda _\varphi w^2+\lambda _4v^2$. After substituting these expressions into $V(h, \phi , S)$, we find the mass terms from the resulting potential. Diagonalizing the mass matrix for the *h* and $\phi$ fields leads to physical states that are linear combinations of the the *h* and $\phi$ fields with mixing angle $\alpha$ given by:

2 $$\begin{aligned} h_1= & {} h\cos {\alpha }-\phi \sin {\alpha } \end{aligned}$$

3 $$\begin{aligned} h_2= & {} \phi \cos {\alpha }+h\sin {\alpha } \end{aligned}$$

with

4 $$\begin{aligned} \sin (2\alpha )= & {} \frac{2\lambda _4vw}{\sqrt{(\lambda _H v^2 - \lambda _\varphi w^2)^2 +4\lambda _4^2 v^2w^2}} \end{aligned}$$

5 $$\begin{aligned} \cos (2\alpha )= & {} \frac{\lambda _\varphi w^2-\lambda _H v^2}{\sqrt{(\lambda _H v^2 - \lambda _\varphi w^2)^2 +4\lambda _4^2 v^2w^2}}, \end{aligned}$$

and the scalar masses given by

6 $$\begin{aligned} m_{h_1}^2= & {} \lambda _H v^2+\lambda _\varphi w^2-\frac{\lambda _\varphi w^2-\lambda _H v^2}{\cos {(2\alpha )}} \end{aligned}$$

7 $$\begin{aligned} m_{h_2}^2= & {} \lambda _H v^2+\lambda _\varphi w^2+\frac{\lambda _\varphi w^2-\lambda _H v^2}{\cos {(2\alpha )}} \end{aligned}$$

8 $$\begin{aligned} m_S^2= & {} \mu _S^2+\lambda _{\varphi \varphi SS} w^2. \end{aligned}$$

For small values of $\alpha$, we identify $h_1$ with the $125~\text {GeV}$ scalar associated with the Standard Model Higgs boson. Because of the mixing, both $h_1$ and $h_2$ act as portals between the Standard Model and the dark matter candidate *S*.

When we scan the parameter space, we will use the physical parameters $m_{h_1}$, $m_{h_2}$, $\alpha$, *v*, and *w*, but the theoretical constraints described below constrain the Lagrangian parameters. We will therefore need the relationships between the physical and the Lagrangian parameters, which are given by

9 $$\begin{aligned} \lambda _H= & {} \frac{1}{4v^2}\left( \left( m_{h_1}^2+m_{h_2}^2\right) -\left( m_{h_2}^2-m_{h_1}^2\right) \cos {2\alpha }\right) \end{aligned}$$

10 $$\begin{aligned} \lambda _\varphi= & {} \frac{1}{4w^2}\left( \left( m_{h_1}^2+m_{h_2}^2\right) +\left( m_{h_2}^2-m_{h_1}^2\right) \cos {2\alpha }\right) \end{aligned}$$

11 $$\begin{aligned} \lambda _4= & {} \frac{\sin {2\alpha }}{4vw}\left( m_{h_2}^2-m_{h_1}^2\right) . \end{aligned}$$

In the following subsections, we examine the theoretical constraints on the Lagrangian parameters.

### Constraints from partial wave unitarity

We start by using partial wave unitarity (PWU) of the $2\rightarrow 2$ scattering amplitudes to constrain the Lagrangian parameters. In the high energy limit, only tree level diagrams involving four-point scalar interactions contribute, as diagrams involving propagators are suppressed by the square of the collision energy. Under these conditions, only the zeroth partial wave amplitude $a_0$ contributes to the $2\rightarrow 2$ amplitudes ${\mathcal {M}}$, so that the constraint $\left| a_0\right| < \frac{1}{2}$ corresponds to ${\mathcal {M}} < 8\pi$. In the high energy limit, we can also use the Goldstone equivalence theorem to replace the gauge bosons with the Goldstone bosons.

There are therefore six fields to consider in the scattering amplitudes: *S*, $\varphi$, and the four Goldstone bosons $\eta ^0$, $\eta ^{0*}$, $\eta ^+$, and $\eta ^-$. The PWU condition must be applied to each of the eigenvalues of the coupled-channel scattering matrix ${\mathcal {M}}$ for all pairs of incoming and outgoing scalar fields. Because the scalar potential is invariant under $SU(2)\times U(1)$ symmetry, the scattering processes conserve electric charge and hypercharge, and can be classified by the total electric charge (*Q*) and hypercharge (*Y*) of the incoming and outgoing states. *S* and $\varphi$ are SM gauge singlets and the Goldstone bosons come from the $SU(2)_L$ doublet with $Y=1$ (where $Q_{em}=T_3 +Y/2$). A symmetry factor of $1/\sqrt{2}$ is included for each pair of identical particles in the initial and final states.

Starting with the $Q=2$ and $Y=2$ quantum numbers, there is only one scattering channel, $\eta ^+\eta ^+\rightarrow \eta ^+\eta ^+$, which leads to the constraint

12 $$\begin{aligned} \left| \lambda _H\right| < 4\pi . \end{aligned}$$

Likewise, the only scattering amplitude for $Q=1$ and $Y=0$ is $\eta ^+{\eta ^0}^* \rightarrow \eta ^+{\eta ^0}^*$, which yields the same constraint.

For $Q=0$ and $Y=1$, there is only the $\eta ^0\varphi \rightarrow \eta ^0\varphi$ scattering amplitude, leading to the constraint

13 $$\begin{aligned} \left| \lambda _4\right| < 4\pi . \end{aligned}$$

Likewise, the only scattering amplitude for $Q=1$ and $Y=1$ is $\eta ^+\varphi \rightarrow \eta ^+\varphi$, which yields the same constraint.

For the $Q=0$ and $Y=0$ quantum numbers, there are five states: $\eta ^0\eta ^{0*}$, $\eta ^+ \eta ^-$, $\varphi \varphi$, $\varphi S$, and *SS*. This results in a $5 \times 5$ scattering matrix consisting of a $4 \times 4$ block and the $\varphi S \rightarrow \varphi S$ channel. The $\varphi S \rightarrow \varphi S$ channel leads to the constraint

14 $$\begin{aligned} \left| \lambda _{\varphi \varphi SS} \right| < 4\pi . \end{aligned}$$

We can partially diagonalize the $4 \times 4$ matrix into a $3 \times 3$ matrix and a diagonal term. The diagonal term leads to the constraint $\left| \lambda _H\right| < 4\pi$. To find the remaining constraints, we diagonalize the $3 \times 3$ matrix by taking its determinant and imposing that the roots of the resulting polynomial satisfy $\left| \text {Roots}\left( p(x)\right) \right| < 8\pi$, where

15 $$\begin{aligned} \begin{aligned} p(x)&=\left( x-3\lambda _S\right) \left( -4\lambda _4^2+\left( x-6\lambda _H\right) \left( x-3\lambda _\varphi \right) \right) \\&\quad -\left( x-6\lambda _H\right) \lambda _{\varphi \varphi SS}^2. \end{aligned} \end{aligned}$$

We follow the procedure of Ref. [83] to which we direct the interested reader for details, and replace the bounds on the roots of *p*(*x*) with the three equivalent conditions:

16 $$\begin{aligned} 16\pi> & {} \left| 6\lambda _H + 3\lambda _\varphi \pm \sqrt{\left( 6\lambda _H-3\lambda _\varphi \right) ^2+16\lambda _4^2}\right| \end{aligned}$$

17 $$\begin{aligned} \lambda _S< & {} \frac{1}{3}\left[ 8\pi +\frac{\left( 6\lambda _H-8\pi \right) \lambda _{\varphi \varphi SS}^2}{\left( 6\lambda _H-8\pi \right) \left( 3\lambda _\varphi -8\pi \right) -4\lambda _4^2}\right] \end{aligned}$$

18 $$\begin{aligned} \lambda _S> & {} \frac{1}{3}\left[ -8\pi +\frac{\left( 6\lambda _H+8\pi \right) \lambda _{\varphi \varphi SS}^2}{\left( 6\lambda _H+8\pi \right) \left( 3\lambda _\varphi +8\pi \right) -4\lambda _4^2}\right] . \end{aligned}$$

Thus, the constraints on the Lagrangian parameters from partial wave unitarity are given by Eqs. (12), (13), (14), (16), (17) and (18).

### Constraints from the Bounded from Below Requirement

We next include constraints on the Lagrangian parameters that ensure that the scalar potential is bounded from below. Because the quartic terms dominate at large field values, this constraint acts on the quartic terms in the potential.

We use the approach described in Ref. [78] (see also Ref. [83]) in which we use a hyperspherical coordinate system replacing the scalar fields by the following parameters:

19 $$\begin{aligned} r= & {} \sqrt{ \left| H \right| ^2 + \varphi ^2 + S^2} \end{aligned}$$

20 $$\begin{aligned} r \sin {\beta }\cos {\gamma }= & {} \left| H \right| ^2\end{aligned}$$

21 $$\begin{aligned} r \sin {\beta }\sin {\gamma }= & {} \varphi ^2\end{aligned}$$

22 $$\begin{aligned} r \cos {\beta }= & {} S^2. \end{aligned}$$

The quartic part of the potential can be then be written as

23 $$\begin{aligned} \frac{r^4}{(1+\tan ^2{\beta })(1+\tan ^2{\gamma })} {\mathbf {x}}^\intercal {\mathbf {A}} {\mathbf {y}} \end{aligned}$$

where

24 $$\begin{aligned} A= & {} \frac{1}{4}\begin{bmatrix} \lambda _S &{} 2\lambda _S&{} \lambda _S \\ 0 &{} 2\lambda _{\varphi \varphi SS} &{} 2\lambda _{\varphi \varphi SS} \\ 4\lambda _H &{}4 \lambda _4 &{} \lambda _\varphi \\ \end{bmatrix} \end{aligned}$$

25 $$\begin{aligned} x= & {} \begin{bmatrix} 1 \\ \tan {\beta } \\ \tan ^2{\beta } \end{bmatrix} \end{aligned}$$

26 $$\begin{aligned} y= & {} \begin{bmatrix} 1 \\ \tan {\gamma } \\ \tan ^2{\gamma } \end{bmatrix}. \end{aligned}$$

Since the prefactor is strictly positive, the requirement for the potential to be bounded from below is that ${\mathbf {x}}^\intercal {\mathbf {A}} {\mathbf {y}}$ be positive. This term can be written as a quadratic in $\tan ^2{\beta }$ with factors themselves quadratics in $\tan ^2{\gamma }$, or vice-versa. Requiring these expressions to be positive leads to the following constraints:

27 $$\begin{aligned} \lambda _H> & {} 0 \end{aligned}$$

28 $$\begin{aligned} \lambda _\varphi> & {} 0\end{aligned}$$

29 $$\begin{aligned} \lambda _S> & {} 0\end{aligned}$$

30 $$\begin{aligned} \lambda _4> & {} -\sqrt{\lambda _H \lambda _\varphi }\end{aligned}$$

31 $$\begin{aligned} \lambda _{\varphi \varphi SS}> & {} -\sqrt{\lambda _\varphi \lambda _S}. \end{aligned}$$

### Constraints from consistency of the potential

With the sign conventions in our potential, for the *H* and $\varphi$ fields to obtain a vev and for *S* to not obtain a vev we require $\mu _H^2 >0$, $\mu _\varphi ^2 >0$, and $\mu _S^2 >0$. This leads to the following three constraints:

32 $$\begin{aligned} \mu _H^2= & {} \lambda _H v^2+\lambda _4 w^2 > 0 \end{aligned}$$

33 $$\begin{aligned} \mu _\varphi ^2= & {} \lambda _\varphi w^2+\lambda _4 v^2 > 0 \end{aligned}$$

34 $$\begin{aligned} \mu ^2_S= & {} m_S^2 - \lambda _{\varphi \varphi SS} w^2 >0. \end{aligned}$$

Imposing these constraints gives the only consistent set of parameters with a DM candidate. Under these conditions, the potential and minima are unique.

## Parameter scan and relic abundance

The model has eight independent parameters. At the Lagrangian level, these parameters are $\lambda _H$, $\lambda _\varphi$, $\lambda _4$, $\lambda _S$, $\lambda _{\varphi \varphi SS}$, $\mu _H$, $\mu _\varphi$, and $\mu _S$. However, it is more transparent to use more physical parameters. We take these to be $m_{h_1}$, $m_{h_2}$, $m_S$, the *h*-$\varphi$ mixing angle $\alpha$, the two vacuum expectation values *v* and *w*, and retain the Lagrangian parameters $\lambda _{\varphi \varphi SS}$ and $\lambda _S$. The relationship between these and the Lagrangian parameters was given by Eqs. (9), (10), (11), (32), (33) and (34).

We identify *v* with the SM Higgs vacuum expectation value and $m_{h_1}$ with the observed 125 GeV scalar mass, leaving six parameters. Of these, $\lambda _S$ is constrained by dark matter self-interaction and Eqs. (17) and (18). When these two constraints are not mutually exclusive, $\lambda _S$ can be set to an arbitrary value that satisfies these constraints without impacting any other quantity of interest. $\lambda _{\varphi \varphi SS}$ directly influences the dark matter annihilation cross section, and we fix its value to give agreement with the measured relic abundance after all other parameters have been fixed. This leaves $m_{h_2}$, $m_S$, $\alpha$, and *w* as free input parameters.

Our procedure is to randomly choose values for $m_{h_2}$, $m_S$, $\alpha$, and *w*. We can limit the allowed range on $\alpha$ using the measured Higgs boson signal strengths to constrain $|\cos \alpha | > rsim 0.97$. This will be checked later by comparing the calculated and measured signal strengths. We typically scan the four parameters by randomly varying *w* and $m_S$ from $1~\text {GeV}$ to $1~\text {TeV}$, $m_{h_2}$ from $100~\text {GeV}$ to $1~\text {TeV}$, and $\alpha$ from $0.969< |\cos \alpha | < 1.0$. We take $\lambda _S=0.2$. We note that we find no qualitative difference in our results or conclusions by increasing the scan range for $m_S$, $m_{h_2}$, and *w* to larger values so that scanning to 1 TeV is sufficient to reveal the characteristics we are exploring.

We then check the resulting Lagrangian parameters against the relevant theoretical constraints. For the parameter sets that pass this test, we use the micrOMEGAs program [84] to search for values of $\lambda _{\varphi \varphi SS}$ that agree with the measured value for the relic abundance of $\varOmega _\text {DM}=0.1200(12) \; h^{-2}$ [85]. We then check the Lagrangian parameters against the remaining theoretical constraints. For those that pass this test, we calculate and compare to experimental measurements the Higgs boson invisible branching ratio, the Higgs boson signal strength, and the dark matter self-interaction cross section. For those parameter points that pass all these constraints, we calculate the indirect detection cross sections for all possible final states and the direct detection cross section using micrOMEGAs [84]. The goal is to see if parameter points that pass all these theoretical and experimental tests are either ruled out or allowed by current limits on direct and indirect detection cross section measurements.

In the following subsections, we describe the details of how we do these calculations.

**Fig. 1:**
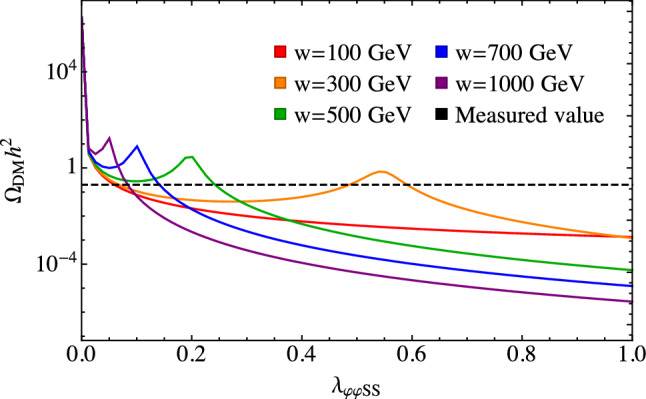
Dark matter relic abundance as a function of $\lambda _{\varphi \varphi SS}$ for $\alpha =0.2$, $m_{h_2}=200~\text {GeV}$, $m_S=300~\text {GeV}$, and for the fixed values of *w* given in the legend. The dashed line is for the measured value of $\varOmega _\text {DM} =0.1200(12)\; h^{-2}$ [85]

### Fitting $\lambda _{\varphi \varphi SS}$ with the relic abundance

We calculate the relic abundance and other DM properties using the micrOMEGAs program [84]. For each set of input parameters, we perform a search by varying $\lambda _{\varphi \varphi SS}$ until we obtain agreement between the calculated value for $\varOmega _\text {DM}$ and the measured value. However, when $m_S > rsim m_{h_2}$, the relic abundance is no longer a monotonic function of $\lambda _{\varphi \varphi SS}$, which complicates the search and can lead to up to three solutions. For these cases, the relic abundance starts by decreasing with increasing $\lambda _{\varphi \varphi SS}$ but then increases again due to a cancellation in the DM annihilation cross sections. This is illustrated in Fig. 1.

**Fig. 2:**
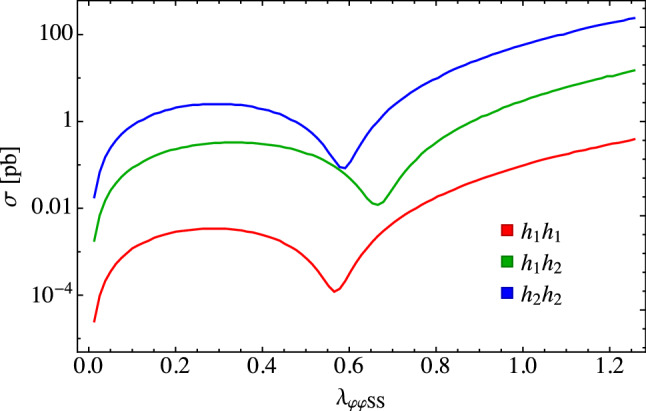
The dark matter annihilation cross section to scalar channels as a function of $\lambda _{\varphi \varphi SS}$ for $\alpha =0.2$, $w=300~\text {GeV}$, $m_{h_2}=200~\text {GeV}$, and $m_S=300~\text {GeV}$, for a center of mass energy of $125~\text {GeV}$. The value of $\lambda _{\varphi \varphi SS}$ where the cross section is a minimum is different for each channel

The cancellation is due to interference between the diagrams contributing to the $SS \rightarrow h_2 h_2$ cross section that, for small $h_1$-$h_2$ mixing angles, occurs at $\lambda _{\varphi \varphi SS} \approx m_S^2/2w^2$. This is a consequence of the Feynman rules for the various vertices entering these matrix elements; the details are presented in Appendix A. As is well known, when the annihilation cross section decreases, the relic abundance increases due to earlier freeze-out. For finite values of the mixing angle, this effect is also present in the $h_1h_2$ and $h_1h_1$ final states, although it occurs at different values of $\lambda _{\varphi \varphi SS}$ for each channel; this can be seen in Fig. 2. While the $h_2h_2$ final state generally dominates because the $h_1h_2$ and $h_1h_1$ are suppressed by factors of $\sin \alpha$ and $\sin ^2\alpha$ respectively, all channels contribute to the relic abundance so that there is no simple formula for the location of the maximum in $\varOmega _\text {DM}$. As a consequence, we use the small mixing angle formula given above to approximate the position of the maxima. While the value of $m_S$ only affects the amplitude of the maxima, $\alpha$ does influence their position, so this formula is not very accurate for large values of $\alpha$. Nonetheless, the formula is an adequate approximation for the local maximum in $\varOmega _\text {DM}$ for the purposes of searching for the values of $\lambda _{\varphi \varphi SS}$ that give the correct relic abundance value $\varOmega _\text {DM}=0.1200(12)\; h^{-2}$ [85].

In general, as pointed out above, there can be up to three values of $\lambda _{\varphi \varphi SS}$ that give $\varOmega _\text {DM}=0.12\; h^{-2}$. We must therefore take some care in our search so that we do not miss one of these solutions. For $m_S<200$ GeV, the maximum is not high enough to yield additional solutions for $\lambda _{\varphi \varphi SS}$. It is therefore sufficient to perform a simple search procedure starting at $\lambda _{\varphi \varphi SS}=0$. From this starting point, we increase $\lambda _{\varphi \varphi SS}$ in small increments until $\varOmega _\text {DM}$ falls below $0.12 \; h^{-2}$, after which we perform a binary search between the last two values of $\lambda _{\varphi \varphi SS}$ until we find a value of $\lambda _{\varphi \varphi SS}$ that yields $\varOmega _\text {DM}=0.12\; h^{-2}$. If this does not occur before $\lambda _{\varphi \varphi SS}$ reaches $4\pi$, the scan is aborted.

For larger values of $m_S$, we determine the position of the maximum in $\varOmega _\text {DM}$ using $\lambda _{\varphi \varphi SS}^\text {max} = m_S^2/2w^2$. If $\varOmega _\text {DM} < 0.12 \; h^{-2}$ for $\lambda _{\varphi \varphi SS}^\text {max}$, there are no additional solutions due to the maximum, and we follow the procedure described above starting at $\lambda _{\varphi \varphi SS}=0$ to determine the unique solution, if it exists.

If $\varOmega _\text {DM} > 0.12\; h^{-2}$, we follow the procedure described above starting at $\lambda _{\varphi \varphi SS}^\text {max}$ to find a solution to the right of the maximum. We repeat this procedure, this time decreasing $\lambda _{\varphi \varphi SS}$ from $\lambda _{\varphi \varphi SS}^\text {max}$ to find a solution to the left of the maximum. If one is found, the procedure is repeated starting from $\lambda _{\varphi \varphi SS}=0$ to find the final solution.

This yields a list of points in the parameter space that give the correct relic abundance. We then check to see that the values of $\lambda _{\varphi \varphi SS}$ satisfy the remaining theoretical constraints given by Eqs. (17), (18), (31) and (34).

Once we have a set of parameters that give the correct relic abundance and satisfy the theoretical constraints, we test them against the experimental constraints.

### Constraints from the Higgs invisible width

The current limits on the invisible width of the $H^0$ boson at 125 GeV is $\text {BR}_\text {invis}<0.26$ at 95% C.L. (ATLAS [86]) and $\text {BR}_\text {invis}<0.19$ at 95% C.L. (CMS [87]). We use the less constraining limit of $\text {BR}_\text {invis}<0.26$ but this has very little effect on our results. Identifying $h_1$ with the $H^0$, the $h_1$ invisible BR is given by

35 $$\begin{aligned} \text {BR}_\text {inv}=\frac{\varGamma _\text {inv}}{\varGamma _\text {total}}=\frac{\varGamma _\text {inv}}{\varGamma _\text {SM}\cos ^2\alpha +\varGamma _\text {inv}} \end{aligned}$$

where $\varGamma _\text {SM} = 4.07$ GeV [85] (see also HDECAY [88]), which is modified by the $h_1$–$h_2$ mixing, $\cos \alpha$. The $h_1 SS$ vertex is $2i\lambda _{\varphi \varphi SS} w \sin {\alpha }$, so that the invisible width is given by

36 $$\begin{aligned} \varGamma _\text {inv}=\frac{\lambda _{\varphi \varphi SS}^2 w^2 \sin ^2{\alpha }}{8\pi m_{h_1}}\sqrt{1-4\frac{m_S^2}{m_{h_1}^2}}. \end{aligned}$$

This constraint eliminates points for $m_S \lesssim m_{h_1}/2$, where the kinematically allowed decay $h_1 \rightarrow SS$ results in a large $\varGamma _\text {inv}$.

### Constraints from the Higgs signal strength

The Higgs signal strength $\mu$ is given by

37 $$\begin{aligned} \mu =\sum _i c_i \omega _i, \end{aligned}$$

where the sum runs over all channels, and where the channel signal strength $c_i$ and the SM channel weight $\omega _i$ are given by

38 $$\begin{aligned} c_i= & {} \frac{\left[ \sigma \times \text {BR}\right] _i}{\left[ \sigma _\text {SM} \times \text {BR}_\text {SM}\right] _i} \end{aligned}$$

39 $$\begin{aligned} \omega _i= & {} \frac{\epsilon _i\left[ \sigma _\text {SM} \times \text {BR}_\text {SM}\right] _i}{\sum _j\epsilon _j\left[ \sigma _\text {SM} \times \text {BR}_\text {SM}\right] _j} \end{aligned}$$

for channel *i* with cross section $\sigma$ ($\sigma _\text {SM}$) and branching ratio $\text {BR}$ ($\text {BR}_\text {SM}$) in the BSM (SM) model and $\epsilon _i$ the experimental efficiency for that channel [89]. For the Standard Model, the Higgs signal strength parameter is $\mu =1$. The current PDG quoted average for the signal strength is $\mu = 1.13 \pm .06$ [85]. In our model, $\mu \le 1$. As such, relative to this best fit point, the 95% C.L. limit is $\mu >0.94$.

For our model, all production channels are modified equally by the $h_1$–$h_2$ mixing, $\cos \alpha$. This leads to a factor of $\sigma _i /\sigma _{\text {SM}i}=\cos ^2{\alpha }$ for the production channels. The decay channels are slightly different as one needs to include the modification of the invisible width in the total width, so that

40 $$\begin{aligned} \frac{\text {BR}_i}{\text {BR}_{\text {SM}i}}=\frac{\varGamma _{\text {SM}}}{\varGamma _{\text {SM}i}}\frac{\varGamma _i}{\varGamma } = \cos ^2{\alpha } \frac{\varGamma _{\text {SM}}}{\varGamma } \end{aligned}$$

where $\varGamma = \varGamma _\text {SM}\cos ^2\alpha +\varGamma _\text {inv}$. Putting it together we obtain

41 $$\begin{aligned} \mu =\cos ^4{\alpha }\frac{\varGamma _\text {SM}}{\varGamma }, \end{aligned}$$

which can be used to apply the constraint $\mu >0.94$. This constraint eliminates parameter points for which $\cos \alpha \lesssim 0.97$, as anticipated. Additionally, when $h_1$ is kinematically allowed to decay to *SS*, the $h_1$ width is significantly larger than the Standard Model value so that the signal strength is altered, also eliminating parameter points.

### Constraints from dark matter self-interaction

At tree level, the strength of dark matter self-interaction is determined by $\lambda _S$ from the quartic coupling and $\lambda _{\varphi \varphi SS}$ from *t*-channel and *s*-channel processes. Once $\lambda _{\varphi \varphi SS}$ is set by the relic abundance, we compare the predicted self-interaction cross section to current limits on $\sigma _\text {DM}$. Constraints from the Bullet Cluster give a limit of $\sigma _\text {SIDM}/m_S < 1.25$ $\hbox {cm}^2/\hbox {g}$ [90], while constraints from colliding galaxies clusters give $\sigma _\text {DM}/m < 0.47$ $\hbox {cm}^2/\hbox {g}$ (95% CL) [91]. We use the tighter constraint of $\sigma _\text {SIDM}/m_S < 0.47$ $\hbox {cm}^2/\hbox {g}$ $\approx 2.2 \times 10^3$ $\hbox {GeV}^{-3}$. However, $\sigma _\text {SIDM}$ only constrains $\lambda _S$, and we have chosen a value that avoids this limit.

### Constraints from indirect detection

Dwarf spheroidal satellite galaxies (dSphs) are typically DM dominated, and so are a good place to study dark matter. We calculated cross-sections for our model using the micrOMEGAs program [84] which outputs $\sigma _\text {ID} v$ at rest. We compared our results to a global analysis by Hoof et al. [92] of DM signals from 27 dwarf spheroidal galaxies using 11 years of observations by Fermi-LAT [93].

**Fig. 3:**
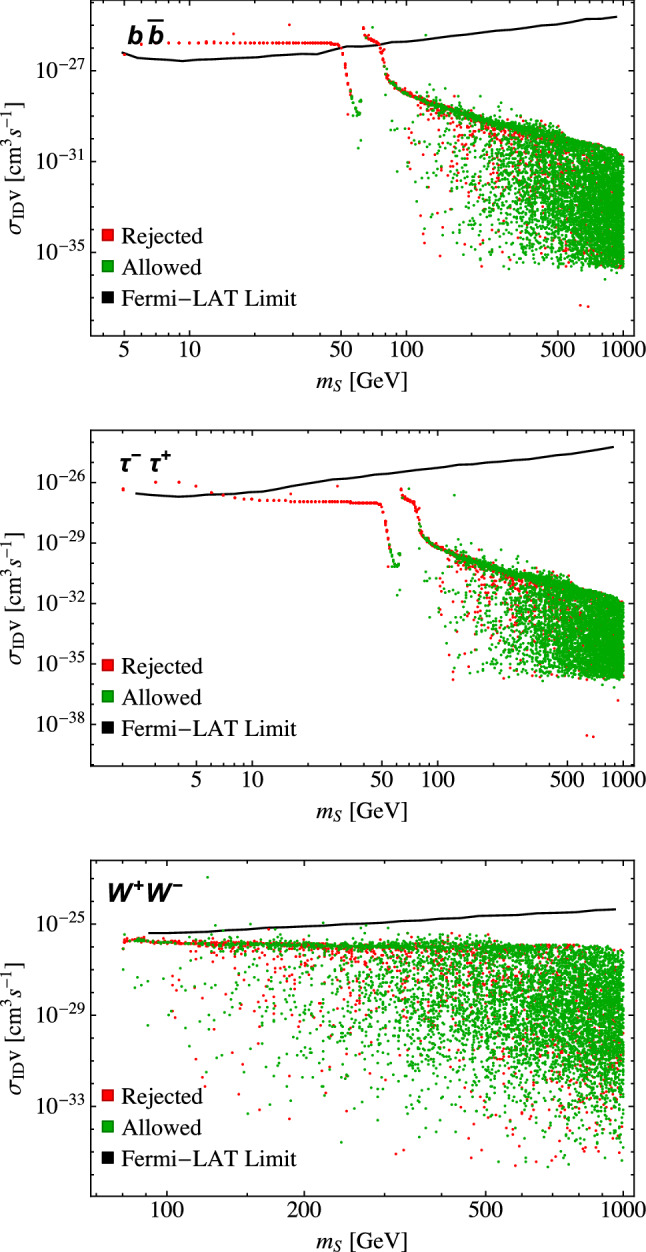
Product of dark matter annihilation cross section and velocity at $v\approx 0$ as a function of the mass of the dark matter candidate *S* for the $b{\bar{b}}$, $\tau ^+\tau ^-$, and $W^+W^-$ final states for the theoretically available points in our scan. Points labeled as “rejected” are points that do not satisfy at least one of the invisible width, Higgs signal strength, or self-interaction constraints

In Fig. 3, we show our results along with the Fermi-LAT limits for the $b{\bar{b}}$, $\tau ^+ \tau ^-$, and $W^+ W^-$ final states. Because $\sigma _\text {ID} v$ is evaluated at threshold, the lower bound is dictated by the kinematic threshold and each plot has a different lower bound. Below $m_S \approx m_{h_1}/2$, the cross sections are relatively insensitive to $m_S$. In this region, the $h_{1,2}SS$ vertices are proportional to $\lambda _{\varphi \varphi SS} w$, which appears in both $\sigma _\text {ID}$ and $\langle \sigma v \rangle$ (which feeds into the relic abundance via the Boltzmann equation [94]). As a consequence, any change in *w* leads to a corresponding change in the value for $\lambda _{\varphi \varphi SS}$ to give the correct relic abundance so that the product $\lambda _{\varphi \varphi SS} w$ remains constant for a given value of $m_S$. In any case, the points for $m_S \lesssim m_{h_1}/2$ are almost always ruled out by $\text {BR}_\text {inv}$ when the decay $h_1 \rightarrow SS$ is kinematically allowed because of the resulting large $\varGamma _\text {inv}$. The dip at $m_S \approx m_{h_1}/2$ is due to the Higgs resonance in the annihilation cross section entering in the calculation of the relic abundance, forcing $\lambda _{\varphi \varphi SS}$ to be small to give the correct relic abundance and resulting in a dip in the indirect detection cross section.

While the indirect detection limits do reject some parameter points for $b{\bar{b}}$ and $\tau ^-\tau ^+$ final states, most of these were already rejected by previous constraints. Only a few points are rejected for the $W^+W^-$ final state, but modest improvements in experimental sensitivity will start ruling out regions of the parameter space allowed by other constraints.

### Constraints from direct detection

Now that all the theoretical constraints and various experimental constraints have been applied to the parameter scan, we turn to our original purpose of confronting the surviving points with the direct detection experimental limits. In this section we compare our parameter points to the limits from the XENON1T experiment [95]. We want to see if patterns emerge with respect to regions in the parameter space where the direct detection cross section is suppressed. We start with an overview of the direct detection cross sections ($\sigma _\text {DD}$) for the scan of parameter points and then examine specific characteristics of the results.

**Fig. 4:**
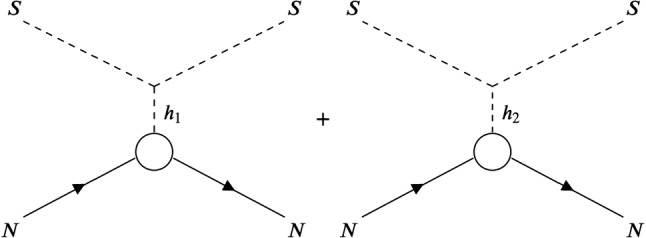
Feynman diagrams for the *t*-channel exchange involved in direct detection, where *N* is the nucleon

In our model, the Higgs boson *t*-channel exchange from a Higgs portal is replaced with *t*-channel exchange of the $h_1$ and $h_2$ which is shown in Fig. 4. The direct detection cross section for scalar DM with a Higgs portal is given by [96]

42 $$\begin{aligned} \sigma _\text {DD} = {1\over {4\pi }} {{M_N^2}\over {(m_S+M_N)^2}} {{ f_N^2 M_N^2}\over {v^2}} \left( {\lambda _{hs}\over { m_h^2}}\right) ^2 \end{aligned}$$

with $m_h$ the Higgs boson mass, $\lambda _{hs}$ the Higgs-scalar DM coupling, $M_N=938.95$ MeV the nucleon mass, and $f_N=0.30$ the Higgs nucleon coupling so that $h_1$ and $h_2$ exchange results in the following substitution

43 $$\begin{aligned} \left( {\lambda _{hs}\over { m_h^2}}\right) ^2\rightarrow & {} \left( { {g_{{h_1} SS}\cos \alpha }\over {m_{h_1}^2}} + { {g_{{h_2}SS}\sin \alpha }\over {m_{h_2}^2}} \right) ^2 \nonumber \\= & {} 4 \cos ^2\alpha \sin ^2 \alpha \lambda _{\varphi \varphi SS}^2 w^2 \left( { {1}\over {m_{h_1}^2}} - {{1}\over {m_{h_2}^2}} \right) ^2, \end{aligned}$$

where we used the relations from Eqs. (48) and (49). One notes the cancellation between the two *t*-channel exchanges and, more importantly, that the direct detection cross section is proportional to $\lambda _{\varphi \varphi SS}^2$ which, as pointed out above, is fitted to give the correct relic abundance.

**Fig. 5:**
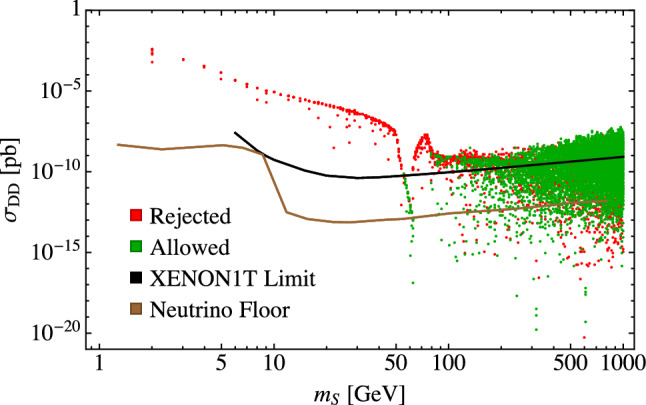
The dark matter direct detection cross section as a function of $m_S$ for the 8148 theoretically allowed points from our scan of 10,000 points. Points labeled as “rejected” are points that do not satisfy at least one of the invisible width, Higgs signal strength, self-interaction, or indirect detection constraints

Figure 5 shows the direct detection cross sections calculated using micrOMEGAs [84] for the 8148 points of the original 10,000 points that passed the theoretical constraints in our parameter scan. The red points were rejected by at least one of the invisible width, Higgs signal strength, dark matter self-interaction, or indirect detection constraints. We remind the reader that, for a given value of $m_S$, we vary $m_{h_2}$, $\cos \alpha$, and *w*. We fit $\lambda _{\varphi \varphi SS}$ to give the correct relic abundance, and since $\lambda _S$ is mainly constrained by the self-interaction cross section, we chose a value that passes this constraint.

In the region below $m_S\approx m_{h_1}/2$, $\sigma _\text {DD}$ is mainly determined by $m_S$ as can be seen from Eq. (42) with slight variations due to the value of $\alpha$, and is largely independent of the other parameters. This is for the same reason as with indirect detection as discussed in Sect. 3.5: the $h_{1,2}SS$ vertices are proportional to $\lambda _{\varphi \varphi SS} w$, which appears in both $\sigma _\text {DD}$ and $\langle \sigma v \rangle$, so that any change in *w* leads to a corresponding change in the value for $\lambda _{\varphi \varphi SS}$ to give the correct relic abundance and the product $\lambda _{\varphi \varphi SS} w$ remains constant for a given value of $m_S$. Likewise, as also discussed in Sect. 3.5, the dip in $\sigma _\text {DD}$ around $m_S \approx m_{h_1}/2$ is due to the Higgs resonance where $\lambda _{\varphi \varphi SS}$ needs to be small to compensate for the enhancement in the *SS* annihilation cross section to obtain the correct relic abundance.

**Fig. 6:**
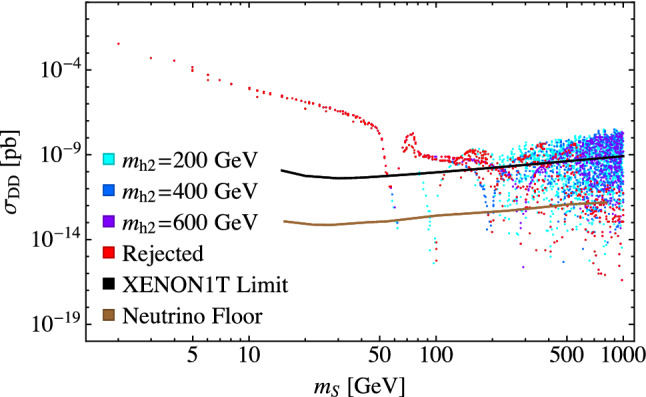
The dark matter direct detection cross section as a function of $m_S$ for random theoretically allowed points with fixed values of $m_{h_2}$. Points labeled as “rejected” are points that do not satisfy at least one of the invisible width, Higgs signal strength, self-interaction, or indirect detection constraints

**Fig. 7:**
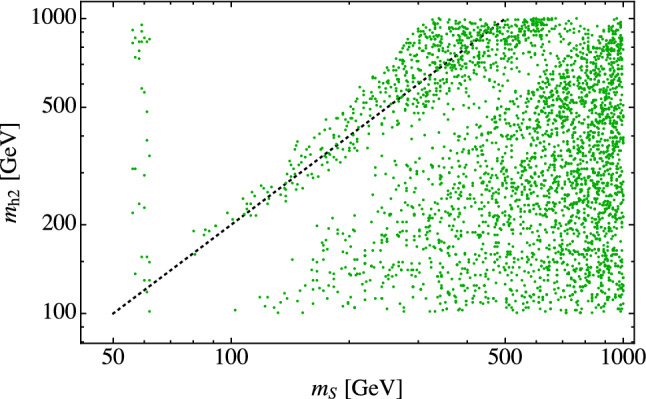
All points allowed by invisible width, Higgs signal strength, self-interaction, indirect detection, and direct detection constraints plotted on the $m_{h_2}$-$m_S$ plane. The dotted line indicates $m_{h_2} = 2 m_S$

The region for $m_S > rsim 80$ GeV shows numerous parameter points not ruled out by direct detection limits. There are two effects contributing to this. The first is due to the resonance effect of the portal scalar when $m_S \approx m_{h_2}/2$, which is analogous to the Higgs resonance effect described above [9, 18, 21]. Near the $h_2$ resonance, the *SS* annihilation cross section increases, requiring a smaller value for $\lambda _{\varphi \varphi SS}$ to obtain the correct relic abundance, resulting in a small direct detection cross section. This is illustrated in Fig. 6 which shows, in addition to the Higgs/$h_1$ resonance, dips in the direct detection cross section at $m_S=100$, 200 and 300 GeV corresponding to $m_{h_2}=200$, 400 and 600 GeV respectively. The linear relationship corresponding to $m_{h_2} \approx 2 m_S$ shows up clearly as a cluster of points along the diagonal in Fig. 7, which plots the parameter points allowed by direct detection on a plot of $m_{h_2}$ vs $m_S$. The cluster of points in the vertical band at $m_S \approx 62.5$ GeV corresponds to the Higgs resonance, and the cluster of points below the diagonal in the bottom right portion of the plot reflects a second effect which we discuss next. The lack of points along $m_S=m_{h_2}$ simply reflects the fact that there are no similar effects in that region.

**Fig. 8:**
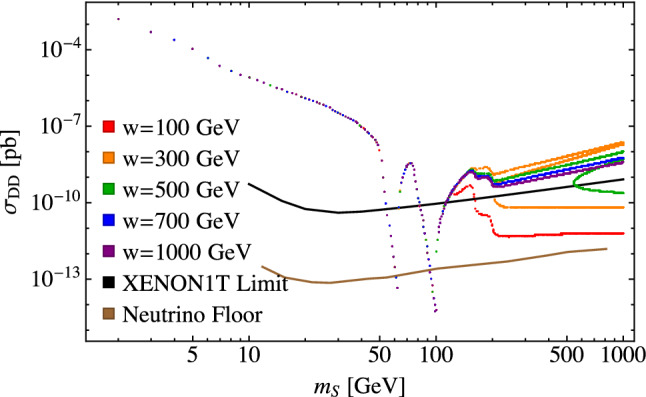
The dark matter direct detection cross section as a function of $m_S$ for theoretically allowed points with $\alpha =0.2$, $m_{h_2}=200~\text {GeV}$, and the fixed values of *w* given in the legend

This second effect results in a big spread of the direct detection cross section and the allowed parameter points, and is more interesting due to non-trivial relationships between the parameters *w* and $\lambda _{\varphi \varphi SS}$ and how this influences the annihilation cross sections as described in Sect. 3. We refer to Fig. 8 to examine the details of this behavior. For $m_S < m_{h_i}$ where $i=1$ or 2, the annihilation cross section is dominated by $SS \rightarrow W^+W^-$ and *ZZ*, while for $m_S > m_{h_i}$ the annihilation cross sections into $h_1$ and $h_2$ become important for achieving the correct relic abundance. In Fig. 8, we see that the resulting direct detection cross section drops at $m_S=m_H=125$ GeV and again at $m_S=m_{h_2}=200$ GeV, the value used for $m_{h_2}$ in this figure. These points correspond to where the $SS\rightarrow h_i h_i$ annihilation channels open up so that a smaller value of $\lambda _{\varphi \varphi SS}$ is needed to achieve the correct relic abundance.

When $m_S > m_{h_i}$, the direct detection cross section in Fig. 8 depends on the value of *w* because it is the product $w \lambda _{\varphi \varphi SS}$ that enters the expressions for the *s*-channel annihilation cross sections for $SS \rightarrow h_i h_j$, where $i,j=1$ or 2. In this situation, as seen in Fig. 1, there can be multiple values of $\lambda _{\varphi \varphi SS}$ that give the correct relic abundance for a given set of the free parameters, $m_{h_2}$, $m_S$, $\alpha$, and *w*, due to the peak in $\varOmega _{DM}$ at $\lambda _{\varphi \varphi SS} \approx m_s^2/2w^2$. This results in the multiple values for the direct detection cross section seen in Fig. 8. Referring to Fig. 1, we can see that this situation only arises for intermediate values of *w*. This is because for small values of *w* the peak shifts to large values of $\lambda _{\varphi \varphi SS}$ where $\varOmega _{DM}$ falls below the observed value, while for large values of *w* the calculated value of $\varOmega _{DM}$ sits above the measured value until after the peak. As such, for small and large values of *w*, there is only one solution for $\lambda _{\varphi \varphi SS}$. The multiple values of $\lambda _{\varphi \varphi SS}$ for intermediate values of *w* result in multiple values for the direct detection cross section, although it should be noted that the additional points with large values of $\lambda _{\varphi \varphi SS}$ are more likely to be inconsistent with direct detection limits.

**Fig. 9:**
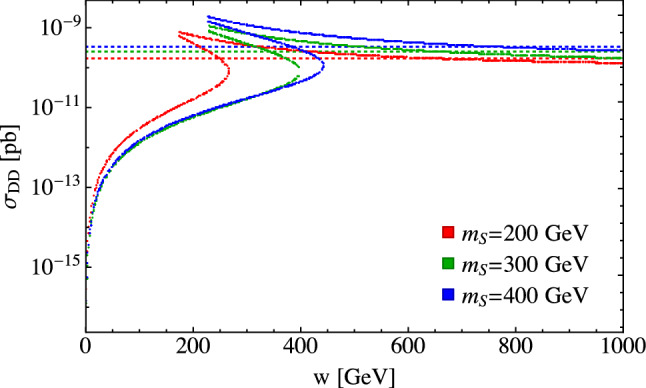
Dark matter direct detection cross section as a function of *w* for theoretically available points with $\alpha =0.1$, $m_{h_2}=200~\text {GeV}$, and the fixed values of $m_S$ given in the legend. The dotted lines correspond to the XENON1T limit for each corresponding value of $m_S$. For each value of $m_S$ shown, there is an intermediate range of *w* values that have no points below the direct detection cross section limit

We can see how the solutions evolve with *w* from a different perspective in Fig. 9, where we plot $\sigma _{DD}$ versus *w* while keeping the other parameters fixed and as usual fitting $\lambda _{\varphi \varphi SS}$ to give the correct relic abundance. The horizontal lines are the XENON1T limits, so points below the lines are allowed and points above are ruled out. The regions of parameter space at both small and large values of *w* are allowed by the direct detection limits. In the intermediate region, starting with small values of *w*, there are multiple values for the direct detection cross sections reflecting the multiple solutions for $\lambda _{\varphi \varphi SS}$ that give the correct relic abundance. In this region, some solutions give rise to large direct detection cross sections that are ruled out by experimental limits while others are allowed. As *w* increases further, we leave the region of multiple solutions and the remaining solutions are ruled out by direct detection limits until eventually they fall below the XENON1T limits. The size of the ruled out region depends on the cancellations of the dark matter annihilation cross sections for the available scalar channels. In our model, when kinematically allowed, the $SS\rightarrow h_2 h_2$ channel dominates. However, this region could be larger for cases where multiple scalar channels are comparable in importance.

### Effect of taking $\lambda _{HHSS} \ne 0$

**Fig. 10:**
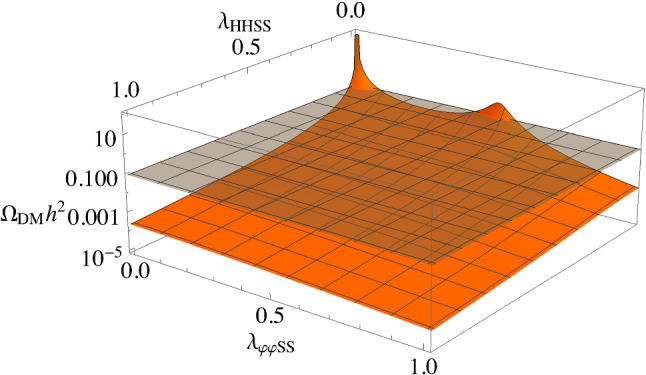
Dark matter relic abundance as a function of $\lambda _{\varphi \varphi SS}$ and $\lambda _{HHSS}$ for $\alpha =0.2$, $m_{h_2}=200~\text {GeV}$, $m_S=300~\text {GeV}$, and $w=300~\text {GeV}$. The grey plane is for the measured value of $\varOmega _\text {DM} =0.1200(12)\; h^{-2}$ [85]

We end this section with some comments on the consequences of not setting $\lambda _{HHSS}$ to zero in Eq. (1). We chose $\lambda _{HHSS} = 0$ to highlight the interplay between parameters of the model, and altering this choice will not affect our conclusions. Allowing $\lambda _{HHSS} \ne 0$ introduces an additional parameter so that for this case it is a linear combination of $\lambda _{\varphi \varphi SS}$ and $\lambda _{HHSS}$ that is fitted to reproduce the observed relic abundance. This gives a family of solutions for these two Lagrangian parameters when keeping the rest of the parameters fixed. This is illustrated in Fig. 10 where the relic abundance is plotted as a function of $\lambda _{\varphi \varphi SS}$ and $\lambda _{HHSS}$ with the other parameters fixed to the same values given in Fig. 1; $\alpha =0.2$, $m_{h_2}=200$ GeV, $m_S=300$ GeV, with the choice $w=300$ GeV. We see that there is now a continuum of solutions, with our choice in this paper corresponding to solutions where $\lambda _{HHSS} = 0$. Rotating from the $\lambda _{HHSS} = 0$ axis to the $\lambda _{\varphi \varphi SS}=0$ axis simply corresponds to another choice of parameters. For the parameters used in Fig. 10, we can obtain the correct relic abundance for a continuum of $\lambda _{HHSS}$ and $\lambda _{\varphi \varphi SS}$ values, but the multi-valueness of $\varOmega _{DM}$ is only present near the $\lambda _{HHSS} = 0$ axis, so that when $\lambda _{HHSS}\ne 0$ the prediction for the direct detection cross section is more straightforward. Thus, taking $\lambda _{HHSS} \ne 0$ does not qualitatively change our results but misses the subtleties and richness of the effects that are discussed in this paper.

## Conclusions

We studied a simple model of scalar DM with a scalar portal that can mix with the SM Higgs. Our purpose was to explore regions of parameter space with a suppressed direct detection cross section for a Higgs portal model. We found that even in this simple model there remains significant regions of parameter space that are not ruled out by direct detection measurements, with many points lying below the neutrino floor. Three of the mechanisms leading to these regions have been discussed previously; a small Higgs-portal mixing angle leading to a small coupling with the DM, the Higgs resonance effect which requires a small DM-portal coupling to compensate for the enhanced DM annihilation cross section due to the Higgs resonance, and the similar effect as a result of the portal resonance.

An additional effect is the result of a heavy DM particle with a lighter portal. This opens up new DM annihilation channels so that the parameters controlling this annihilation need to compensate, resulting in a smaller direct detection cross section. For certain regions of the parameter space, destructive interference between diagrams leads to multiple solutions for the DM-portal couplings, resulting in a spread of allowed parameter points. We therefore find, contrary to common lore, that even in a very simple model of DM there are sizeable regions of parameter space that are still allowed by direct detection limits.

## Figures and Tables

**Fig. 11:**
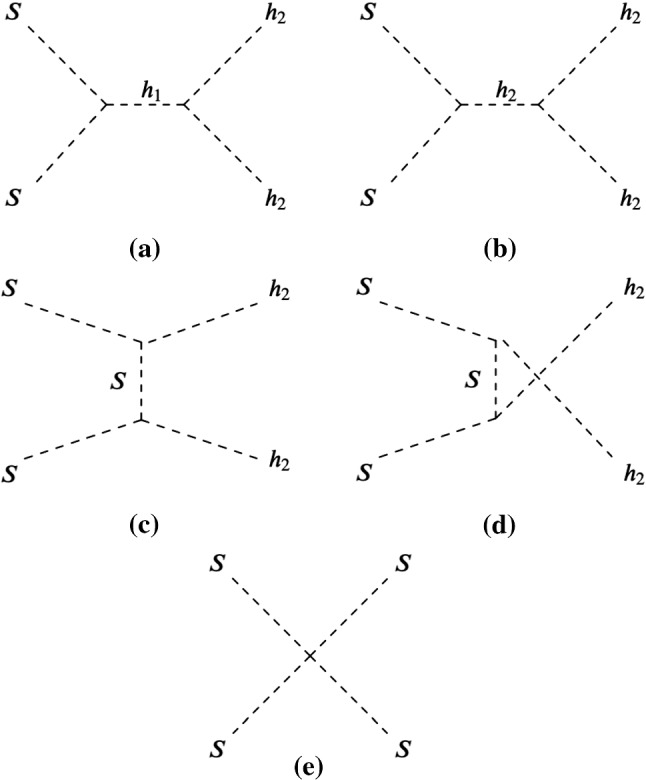
Feynman diagrams contributing to the $SS\rightarrow h_2h_2$ process

## Data Availability

This manuscript has no associated data or the data will not be deposited. [Authors’ comment: This is a theory calculation so there is no data associated with it.]

## Appendix A: Interference in the dark matter annihilation amplitude

The model presented in Sect. 2 features a dark matter candidate *S* which couples only to the other two physical scalars $h_1$ and $h_2$, giving three annihilation channels: $SS\rightarrow h_1h_1$, $SS\rightarrow h_1h_2$, and $SS\rightarrow h_2h_2$. At tree-level, each of these channels features the five diagrams shown in Fig. 11: two *s*-channels with $h_1$ and $h_2$ mediators, *t*- and *u*-channels with an *S* mediator, and a quartic vertex.

The relevant vertices are given by

44 $$\begin{aligned} g_{h_1h_1h_1}= & {} 6i(\lambda _H v c_\alpha ^3 - \lambda _4 w c_\alpha ^2 s_\alpha + \lambda _4 v c_\alpha s_\alpha ^2 \nonumber \\&- \lambda _\varphi w s_\alpha ^3) \end{aligned}$$

45 $$\begin{aligned} g_{h_1h_1h_2}= & {} 2i(\lambda _4 w c_\alpha ^3 - \left( 2\lambda _4 -3\lambda _H\right) v c_\alpha ^2 s_\alpha \nonumber \\&- \left( 2\lambda _4 -3\lambda _\varphi \right) w c_\alpha s_\alpha ^2 + \lambda _4 v s_\alpha ^3)\end{aligned}$$

46 $$\begin{aligned} g_{h_1h_2h_2}= & {} 2i(\lambda _4 v c_\alpha ^3 + \left( 2\lambda _4 -3\lambda _\varphi \right) w c_\alpha ^2 s_\alpha \nonumber \\&- \left( 2\lambda _4 -3\lambda _h\right) v c_\alpha s_\alpha ^2 - \lambda _4 w s_\alpha ^3) \end{aligned}$$

47 $$\begin{aligned} g_{h_2h_2h_2}= & {} 6i(\lambda _\varphi w c_\alpha ^3 + \lambda _4 v c_\alpha ^2 s_\alpha + \lambda _4 w c_\alpha s_\alpha ^2 \nonumber \\&+ \lambda _H v s_\alpha ^3) \end{aligned}$$

48 $$\begin{aligned} g_{h_1SS}= & {} - 2i \lambda _{\varphi \varphi SS} w s_\alpha \end{aligned}$$

49 $$\begin{aligned} g_{h_2SS}= & {} 2i \lambda _{\varphi \varphi SS} w c_\alpha \end{aligned}$$

50 $$\begin{aligned} g_{h_1h_1SS}= & {} 2i \lambda _{\varphi \varphi SS} s_\alpha ^2 \end{aligned}$$

51 $$\begin{aligned} g_{h_1h_2SS}= & {} -2i \lambda _{\varphi \varphi SS} c_\alpha s_\alpha \end{aligned}$$

52 $$\begin{aligned} g_{h_2h_2SS}= & {} 2i \lambda _{\varphi \varphi SS} c_\alpha ^2, \end{aligned}$$

where $c_\alpha =\cos {\alpha }$ and $s_\alpha =\sin {\alpha }$. In the limit $\alpha \rightarrow 0$, using Eqs. (10) and (9), these vertices become

53 $$\begin{aligned} g_{h_1h_1h_1}= & {} 3i\frac{m_{h_1}^2}{v} \end{aligned}$$

54 $$\begin{aligned} g_{h_2h_2h_2}= & {} 3i\frac{m_{h_2}^2}{w} \end{aligned}$$

55 $$\begin{aligned} g_{h_2SS}= & {} 2i\lambda _{\varphi \varphi SS}w \end{aligned}$$

56 $$\begin{aligned} g_{h_2h_2SS}= & {} 2i\lambda _{\varphi \varphi SS}, \end{aligned}$$

with all other couplings going to 0, effectively decoupling $h_1$ from the other scalars.

Under this approximation, the amplitudes of $SS\rightarrow h_1h_1$ and $SS\rightarrow h_1h_2$ vanish, and the amplitude of $SS\rightarrow h_2h_2$ is given by

57 $$\begin{aligned} {\mathcal {M}}_{SS\rightarrow h_2h_2}= & {} g_{h_2h_2SS}-\frac{ig_{h_2h_2h_2}g_{h_2SS}}{s-m_{h_2}^2}\nonumber \\&-\frac{ig_{h_2SS}^2}{t-m_S^2}-\frac{ig_{h_2SS}^2}{u-m_S^2} \end{aligned}$$

58 $$\begin{aligned}\approx & {} 4i \lambda _{\varphi \varphi SS} - 8i\frac{w^2\lambda _{\varphi \varphi SS}^2}{m_S^2}, \end{aligned}$$

where we used a threshold approximation to set the Mandelstam variables to $s=\left( 2m_{h_2}\right) ^2$ and $t=u=0$. The resulting amplitude is zero at both $\lambda _{\varphi \varphi SS}=0$ and $\lambda _{\varphi \varphi SS}=m_S^2/2w^2$.

## Footnotes

1 References [12, 21, 25] noted a similar effect when $m_S > m_{h_1,h_2}$ although the details differ from those presented here.
